# Freshwater microalgae (*Schizochytrium sp*.) as a substitute to fish oil for shrimp feed

**DOI:** 10.1038/s41598-019-41020-8

**Published:** 2019-04-16

**Authors:** Kristy M. Allen, Habte-Michael Habte-Tsion, Kenneth R. Thompson, Keith Filer, James H. Tidwell, Vikas Kumar

**Affiliations:** 1 Division of Aquaculture, College of Agriculture, Food Science and Sustainable Systems, Kentucky State University, 103 Athletic Dr, Frankfort, KY 40601 USA; 2 Aquaculture Research Center, Alltech, 3031 Catnip Hill Road, Nicholasville, KY 40356 USA; 3 Aquaculture Research Institute, Department of Animal and Veterinary Science, University of Idaho, 2000 W Sixth Street, Moscow, ID 83844 USA

## Abstract

Micro-algae, *Schizochytrium* sp., is rich source of docosahexaenoic acid, DHA (66%-lipid with 27%-DHA). Eight nutritionally balanced-diets were formulated: diet 1 (control) consisted of only fish oil (FO); diets 2 and 3 had increasing amounts of algae-meal and soybean oil (SBO) at the expense of FO; diet 4 consisted of a combination of algae meal (37-g/kg), SBO (21-g/kg), and linseed oil (LSO) at 4-g/kg each; diet 5 had microalgae meal at 50-g/kg and equal amounts of LSO and SBO at 8-g/kg; diets 6 and 7 contained equal amounts of algae-meal at 62-g/kg, but with LSO or SBO added at 8-mg/g, respectively; diet 8 contained only algae-meal at 75-mg/g. Growth and feeding efficiencies of *L. vannamei* were not significantly different among treatments. Fatty acid composition of muscle generally reflected that of the diet. The amount of muscle sub-epidermal adipose tissue was significantly higher for shrimp fed diets 3 and 7, while intestinal lipase was significantly higher in shrimp fed diets 7 and 8. Muscle lipid peroxidation was unaffected by the dietary treatments, although antioxidant activities were significantly higher in shrimp fed diet 7 compared to those fed diet 1. Overall algal-meal can completely replace the FO in shrimp feed.

## Introduction

Aquaculture is currently growing 10% faster than capture fisheries and generated US $160.2 billion in sales in 2015^1^. Currently, the world produces nearly 44 million tonnes of finfish products and nearly 3 million tonnes of crustaceans contributing at inland aquaculture farms (Food and Agriculture Organization of the United Nations, FAO)^1^. Among crustaceans produced in aquaculture, the Pacific white shrimp (*Litopenaeus vannamei*) is produced in the largest amount by far, which can be attributed to their fast growth, high market value, and higher resistance to disease^2^. As shrimp aquaculture has increased, demand for marine based ingredients, such as fish oil (FO) has also risen. This resource, however, is becoming more expensive, which is leading to investigations in identifying suitable alternatives without jeopardizing finfish and crustacean health^3,4^. Among potential FO alternatives, oils from thraustochytrid microalgae, such as *Crypthecodinium* sp. and *Schizochytrium* sp., have been recognized as promising ingredients in aquafeeds due to their high long chain polyunsaturated fatty acids (LC-PUFA) content and the fact that they result in similar or better growth performance compared to FO in various aquatic animals^5–10^. For example, channel catfish (*Ictalurus punctatus*) fed diets with increasing amounts of dried *Schizochytrium* meal (0 to 20 g/kg) at the expense of catfish offal oil had significantly increased weight gain when fed at 10 or 15 g/kg^8^. Similarly, when *Schizochytrium* was used as FO replacer for Atlantic salmon parr (*Salmo salar*) feed, growth was not compromised, and the microalgae oil was as digestible as FO also^7^.

The inclusion of dietary microalgae oil in the diets of Pacific white shrimp has also shown great promise^6,10–13^. Complete replacement of FO with different combinations of *Schizochytrium* oil, *Mortierella* oil, soybean oil (SBO), and flax oil in plant protein-based diets resulted in similar growth and survival to a commercial (control) diet^6^. A subsequent study showed that a combination of dietary *Schizochytrium* and *Mortierella* oil improved growth in *L. vannamei* slightly compared to a FO-based diet^11^. Similarly, the sole use of dietary *Schizochytrium* meal resulted in similar growth in *L. vannamei* as FO without compromising survival or digestive intestinal and hepatopancreatic enzyme activity^10,12^. In addition, Nonwachai *et al*.^13^ found that dietary *Schizochytrium* meal enhanced innate immunological responses in *L. vannamei*, including phagocytic activity, antioxidant enzyme activity, and total hemocyte counts, as well as their resistance to the marine pathogen, *Vibrio harveyi*. These findings, along with data that suggest microalgae meal often leading to a high LC-PUFA content in *L. vannamei* fed either plant oil-based- or FO-based diets^10,11^, indicates this sustainable resource rich in omega-3 fatty acids can provide more benefits to both the fish and human consumer.

Currently, the implications of using dietary microalgae meal on parameters associated with lipid metabolism such as lipase activity, total cholesterol, and sub-epidermal adipose tissue remain unexplored. The aim of this study was to evaluate the effects of replacing dietary FO with *Schizochytrium* meal alone or in combination with SBO and/or LSO on survival, growth, muscle fatty acid composition, muscle antioxidant status and intestinal lipase activity of Pacific white shrimp, *L. vannamei*.

## Materials and Methods

### Preparation of the algal meal and experimental diets

The fermented algae, *Schizochytrium* sp., was used in the current study (Source: Alltech Lexington, KY, USA). The fermented algae were continuously centrifuged (5,000 g) to obtain the solid thereafter sprayed dried yielding a fine algae powder. Kochiwa spray dryer [using centrifugal atomization (4,500 g) at an inlet temperature (140–150 °C), central chamber (130–135 °C) and an outlet temperature of (80–85 °C)] were used to prepare the spray-dried powder. It took 6–8 sec to form the algae powder after injection. The proximate composition was measured according to standard Association of Official Analytical Chemists methods^14^, which showed this meal contained a lipid content of 66%. The proximate composition of ingredients such as SBM, wheat meal, and wheat gluten were similarly measured. A total of eight iso-nitrogenous diets (containing 40% protein) and iso-energetic (9.0% lipid) were formulated to contain increasing amounts of the algae meal (containing 660 g/kg extractable lipid; Table 1). Regardless of the diet, fishmeal (FM) and soybean meal (SBM) were the dominant protein sources and were added to all experimental diets at 250 and 397 g/kg, respectively. The amount of crude protein, lipid, vitamins, and minerals were formulated to meet the requirements of penaeid shrimp^15^. In addition, cholesterol, choline chloride, and lecithin were included in all diets to satisfy the requirements of *L. vannamei*^16,17^. The control diet (diet 1) had 6% FO as the main lipid source, which is similar to the amount used in commercial diets (Table 1). Diets 2 and 3 had increasing amounts of algae meal and SBO at the expense of FO. FO was completely replaced in diet 4 with a combination of algae meal, SBO, and LSO. Diets 5–8 also contained no FO, but had different combinations of lipids: diet 5 had equal amounts of LSO and SBO at 8 g/kg; diets 6 and 7 contained equal amounts of algae meal, but with LSO or SBO added at 8 g/kg, respectively; diet 8 contained only algae meal as the only added lipid source, with the highest inclusion at 75 g/kg. Wheat meal was adjusted in the experimental diets to compensate for the small amount of crude protein in the algae meal (Table 1). Meanwhile, *Schizochytrium* meal is low in EPA, but can be synthesized by some aquatic animals from the precursor, ALA. Therefore, LSO, which is high in ALA, was added in diets 4–6 to determine whether this could result in high EPA levels in the shrimp. Dried feed ingredients were thoroughly grinded and mixed in mixer (Hobart A200, Hobart, Troy, OH, USA) to prepare the shrimp diets. Followed by warm water addition to maintain the 35% moisture and mixed for another 10 min. This dough of each diet was passed via feed pelletizer (meat grinder, Glen Mills Inc., Clifton, NJ, USA) with a 2-mm die followed by air dried for 24 hours. Long strands of diets were pulverized with grinder (Glen Mills Inc., Clifton, NJ, USA) and obtained 2 mm pellet size via using sieve. After sieving, appropriate amounts of LSO, SBO, and FO were manually added to inhibit the damage of highly unsaturated fatty acids during pelletizing. After the diets were dried, chemical composition including fatty acids of diets was determined^14^ (Table 1).

**Table 1:** Ingredient and proximate composition (g/kg) of the experimental diets.

Ingredients	Experimental diets							
	1	2	3	4	5	6	7	8
Fishmeal	250	250	250	250	250	250	250	250
Soybean meal	397	397	397	397	397	397	397	397
Wheat meal	180	176	172	168	164	159	159	156
Wheat gluten	80	80	80	80	80	80	80	80
Fish oil	50	25	13	0	0	0	0	0
Linseed oil	0	0	0	4	8	8	0	0
Soybean oil	0	17	21	21	8	0	8	0
Algae meal (66% oil)	0	12	25	37	50	62	62	75
Cholesterol	2	2	2	2	2	2	2	2
Lecithin	5	5	5	5	5	5	5	5
DCP	15	15	15	15	15	15	15	15
MgSO_4_	6	6	6	6	6	6	6	6
KH_2_PO_4_	8	8	8	8	8	8	8	8
Vitamin C	1	1	1	1	1	1	1	1
Vitamin premix^a^	4	4	4	4	4	4	4	4
Mineral premix^b^	1	1	1	1	1	1	1	1
Choline chloride	1.5	1.5	1.5	1.5	1.5	1.5	1.5	1.5
*Proximate composition (g/kg dry matter)*								
Moisture	136	116	123	109	121	122	117	117
Crude protein	407	384	381	377	385	389	392	396
Crude lipid	82	85	85	84	87	90	94	89
Crude ash	108	109	112	109	108	111	108	110

^a^Provides per kg of diet: retinyl acetate (vitamin A), 3000 IU; cholecalciferol (vitamin D), 2400 IU; all-rac-α-tocopheryl acetate (vitamin E), 60 IU; menadione sodium bisulfite (vitamin K), 1.2 mg; ascorbic acid monophosphate (49% ascorbic acid, vitamin C), 120 mg; cyanocobalamine (vitamin B12), 0.024 mg; d-biotin, 0.168 mg; choline chloride, 1200 mg; folic acid, 1.2 mg; niacin, 12 mg; d-calcium pantothenate, 26 mg; pyridoxine, HCl, 6 mg; riboflavin, 7.2 mg; thiamin. HCl, 1.2 mg.

^b^Provides per kg of diet: sodium chloride (NaCL, 3%Na, 61%CL), 3077 mg; ferrous sulfate (FeSO_4_·7H_2_O, 20% Fe), 65 mg; manganese sulfate (MnSO4, 36% Mn), 89 mg; zinc sulfate (ZnSO4·7H2O, 40% Zn), 150 mg; copper sulfate (CuSO4.5H2O, 25% Cu), 28 mg; potassium iodide (KI, 24% K, 76% I), 11 mg; celite AW521 (acid-washed diatomaceous earth silica, 1000 mg).

### Experimental animals and experimental system

Post larvae of Pacific white shrimp (*Litopenaeus vannamei*) were purchased from the private shrimp hatchery (Shrimp Improvement Systems, LLC, Islamorada, FL, USA) and gradually acclimated to a shrimp nursery system at the department of aquaculture, Kentucky State University, Frankfort, KY, USA. A 1,000-L rearing tank (used as a recirculating nursery system) connected to a solid settling tank and a matured biological filter (Red-Ewald, Karnes City, TX, USA) to maintain optimum water parameter conditions for shrimp larvae. Artificial seawater was made by mixing Crystal Sea Marine salt mix (Marine Enterprises International, LLC, Baltimore, MD) with dechlorinated municipal water to a salinity of 34 parts per thousand (ppt). The rearing tanks were provided gentle aeration (ceramic diffusers). Gradually salinity was decreased to 27–28 ppt by addition of freshwater over two weeks acclimation period and were fed commercially available shrimp diets (Zeigler Brothers Inc.).

A total of 600 juvenile shrimp (3.15 ± 0.08 g) were individually weighed, divided into 24 groups consisting of 25 shrimp each, and placed into 110-L glass aquaria. After stocking the shrimp, the eight treatment diets were randomly allocated in triplicate. The aquaria were connected to a 2000-L mechanical and biological propeller-washed bead filtration system (Red-Ewald, Karnes City, TX, USA) at a salinity of 27–28 ppt. Each aquarium was supplied with seawater at a rate of 4.0 L/min and optimum aeration was provided by single 12-inch air diffusers by Rotron blower (Ametek, Kent, OH, USA).

Throughout the experiment, the shrimp were fed 5% of their total body weight daily, which was equally divided and fed at 08:00, 11:30, 15:30, and 18:00 hr. The amount of feed given was considered and accustomed every four weeks based on biomass evaluations after measuring the total weight of shrimp in each tank; the quantity of feed fed each day was noted per aquarium. Shrimp were fed total eight diets (1 control and 7 experimental diets) for 12 weeks period.

Throughout the feeding trial, the water temperature was maintained at approximately 29 °C by an immersion heater. Salinity was maintained by adding fresh water to replace water loss from evaporation or additional salt to replace losses due to filter flushing. The pH, temperature, salinity, and dissolved oxygen were measured once per day using a Hydrolab Quanta meter (Hydrolab Corporation, Loveland, CO). The shrimp were subjected to a photoperiod of 12 h:12 h light: dark cycle by overhead fluorescent ceiling lights. In the morning and evening, all aquaria were siphoned daily to remove uneaten feed and feces. The total ammonia nitrogen (TAN) and nitrite-N (NO_2_-N) concentrations were measured once per week on a Hach DR 3800 spectrophotometer according to the manufacturer instructions (Hach, USA). If the TAN or NO_2_-N exceeded 0.5 mg/L, a partial water exchange was performed.

### Sampling and analysis

After 12 weeks, shrimp were harvested from each aquarium; bulk weighed and counted to calculate growth rates, feeding efficiencies, and survival using the following equations:

$${\rm{Body}}\,{\rm{mass}}\,{\rm{gain}}\,({\rm{BMG}};\, \% )=[({\rm{Final}}\,{\rm{body}}\,{\rm{mass}}-{\rm{initial}}\,{\rm{body}}\,{\rm{mass}})/{\rm{Initial}}\,{\rm{body}}\,{\rm{mass}}]\times 100$$

$${\rm{Feed}}\,{\rm{conversion}}\,{\rm{ratio}}\,({\rm{FCR}})={\rm{dry}}\,{\rm{feed}}\,{\rm{fed}}\,{\rm{in}}\,{\rm{g}}/{\rm{body}}\,{\rm{mass}}\,{\rm{gain}}\,{\rm{in}}\,{\rm{g}}$$

$${\rm{Protein}}\,{\rm{efficiency}}\,{\rm{ratio}}\,({\rm{PER}})={\rm{body}}\,{\rm{mass}}\,{\rm{gain}}\,{\rm{in}}\,{\rm{g}}/{\rm{crude}}\,{\rm{protein}}\,{\rm{fed}}\,{\rm{in}}\,{\rm{g}}$$

$$\begin{array}{rcl}{\rm{Protein}}\,{\rm{productive}}\,{\rm{value}}\,({\rm{PPV}}, \% ) & = & [({\rm{final}}\,{\rm{shrimp}}\,{\rm{body}}\,{\rm{protein}}\,{\rm{in}}\,{\rm{g}}\\  &  & -\,{\rm{initial}}\,{\rm{shrimp}}\,{\rm{body}}\,{\rm{protein}}\,{\rm{in}}\,{\rm{g}})\\  &  & /{\rm{total}}\,{\rm{protein}}\,{\rm{consumed}}\,{\rm{in}}\,{\rm{g}}]\times 100\end{array}$$

$$\begin{array}{ccc}{\rm{L}}{\rm{i}}{\rm{p}}{\rm{i}}{\rm{d}}\,{\rm{p}}{\rm{r}}{\rm{o}}{\rm{d}}{\rm{u}}{\rm{c}}{\rm{t}}{\rm{i}}{\rm{v}}{\rm{e}}\,{\rm{v}}{\rm{a}}{\rm{l}}{\rm{u}}{\rm{e}}\,({\rm{L}}{\rm{P}}{\rm{V}};\,{\rm{ \% }}) &  & [({\rm{f}}{\rm{i}}{\rm{n}}{\rm{a}}{\rm{l}}\,{\rm{b}}{\rm{o}}{\rm{d}}{\rm{y}}\,{\rm{l}}{\rm{i}}{\rm{p}}{\rm{i}}{\rm{d}}\,{\rm{i}}{\rm{n}}\,{\rm{g}}\\  &  & -\,{\rm{i}}{\rm{n}}{\rm{i}}{\rm{t}}{\rm{i}}{\rm{a}}{\rm{l}}\,{\rm{b}}{\rm{o}}{\rm{d}}{\rm{y}}\,{\rm{l}}{\rm{i}}{\rm{p}}{\rm{i}}{\rm{d}}\,{\rm{i}}{\rm{n}}\,{\rm{g}})\\  &  & /{\rm{t}}{\rm{o}}{\rm{t}}{\rm{a}}{\rm{l}}\,{\rm{c}}{\rm{r}}{\rm{u}}{\rm{d}}{\rm{e}}\,{\rm{l}}{\rm{i}}{\rm{p}}{\rm{i}}{\rm{d}}\,{\rm{c}}{\rm{o}}{\rm{n}}{\rm{s}}{\rm{u}}{\rm{m}}{\rm{e}}{\rm{d}}\,{\rm{i}}{\rm{n}}\,{\rm{g}}]\times 100\end{array}$$

After weighing and counting, shrimp were anesthetized in an ice bath (0–4 °C) and samples were collected and stored at −20 °C for further analysis. Five shrimps from each aquarium were used for whole-body proximate composition according to AOAC^14^. Samples of hepatopancreas, intestine and tail muscle were collected from the remaining shrimp and stored frozen (−80 °C) until further analysis.

### Lipid composition of diets and muscle, antioxidant and lipid degradation enzymes

Diets (Table 2) and muscle samples from five shrimp in each tank were analyzed for their fatty acid composition by quantitative gas chromatography (utilizing C23:0 as an internal standard). Total cholesterol from the muscle was analyzed using a colorimetric total cholesterol assay kit according to manufacturer instructions (Cell Biolabs Inc., San Diego, CA). Absorbance was read on a Synergy HTX Multi-Mode Reader (BioTek, Instruments, Inc., Winooski, VT). Muscle samples (with digesta) from six shrimps in each replicate were immediately stored at −80 °C. For analysis, the samples were thawed in an ice bath and homogenized using an ultrasonic processor (Cole Parmer Scientific Experts, Vernon Hills, IL, USA) for 5 min in an ice bath. The samples were then placed in a 1.5 ml microcentrifuge tube (Eppendorf, Hamburg, Germany) with phosphate buffered saline (PBS; Fisher Bioreagents, Fair Lawn, NJ, USA) and cell lysis solution (Promega Corporation, Madison, WI, USA).

**Table 2:** Fatty acids composition (% of total lipid) of the experimental diets.

Fatty acids	Experimental diets							
	1	2	3	4	5	6	7	8
C8	2.81	4.03	2.35	2.08	2.86	3.20	3.17	3.28
C10	0.94	0.95	0.41	0.35	0.36	0.36	0.36	0.36
C11	0.66	0.65	0.29	0.29	0.29	0.29	0.29	0.29
C12	0.34	0.31	0.18	0.19	0.21	0.22	0.22	0.22
C13	0.24	0.24	0.24	0.24	0.24	0.23	0.24	0.24
C14	3.26	2.95	2.96	2.65	3.06	3.56	3.44	3.73
C15	0.26	0.32	0.40	0.47	0.58	0.70	0.68	0.76
C16	12.64	45.32	18.75	20.48	24.34	28.10	28.25	30.90
C17	0.00	0.14	0.17	0.21	0.24	0.27	0.27	0.29
Cis-10-C17	0.52	0.44	0.40	0.33	0.33	0.36	0.35	0.35
C18	2.16	2.27	2.38	2.19	2.11	2.15	2.14	1.97
C20	0.09	0.11	0.13	0.14	0.14	0.15	0.16	0.15
C23	19.97	19.69	19.79	19.61	19.76	19.69	19.70	19.87
C24	0.74	0.65	0.62	0.55	0.56	0.61	0.60	0.60
C16:1n-7	4.69	3.52	2.81	1.84	1.81	2.06	2.00	1.99
C18:1n-9	11.69	10.76	10.72	7.64	6.21	4.60	5.39	3.84
C18:2n-6	10.47	16.60	10.21	17.19	13.76	8.63	12.77	9.22
C18:3n-3	1.55	2.40	2.89	5.92	5.48	5.62	1.96	1.32
C20:1n-9	5.29	3.00	2.00	0.32	0.29	0.27	0.29	0.26
C20:3n-3	0.50	0.44	0.57	0.60	0.68	0.79	0.75	0.80
C20:5n-3	7.02	4.95	4.08	2.56	2.34	2.79	2.42	2.67
C22:6n-3	4.73	5.59	7.17	8.42	10.77	13.34	12.87	14.72
*Major groups*								
Σ SFA	44.63	78.07	49.07	49.78	55.08	59.89	59.87	63.01
Σ MUFA	21.67	17.28	15.53	9.80	8.31	6.93	7.68	6.09
Σ n-6	10.47	16.60	10.21	17.19	13.76	8.63	12.77	9.22
Σ n-3	13.80	13.38	14.71	17.50	19.27	22.54	8.00	19.51
Σ LC-PUFA	12.25	10.98	11.82	11.58	13.79	16.92	16.04	18.19
n-3/n-6	1.31	1.22	1.44	1.02	1.40	2.61	0.63	2.12

SFA, saturated fatty acids = sum C8 to C24.

MUFA, monounsaturated fatty acids = C16:1n-7 + C18:1n-9 + C20:1n-9.

n-6 PUFA, polyunsaturated fatty acids = C18:2n-6.

n-3 PUFA, polyunsaturated fatty acids = C18:3n-3 + C20:3n-3 + C20:5n-3 + C22:6n-3.

LC-PUFA, long chain polyunsaturated fatty acids = C20:3n-3 + C20:5n-3 + C22:6n-3.

The activities of lipase, catalase (CAT), and superoxide dismutase (SOD), and the contents of lipid peroxide and adipogenesis were analyzed using a Synergy HTX Multi-Mode Reader (BioTek, Instruments, Inc., Winooski, VT, USA). Intestinal lipase activity was measured using a QuantiChrom lipase assay kit (DLPS-100; BioAssay Systems, Hayward, CA, USA) according to manufacturer instructions; absorbance was read at 412 nm. The CAT and SOD activities in the tail muscle were measured using commercial test kits (Cayman Chemical, Ann Arbor, MI, USA); absorbance was read at 540 nm and 460 nm, respectively. Lipid peroxide in tail muscle was measured by analyzing the malondialdehyde (MDA) level using a colorimetric/fluorometric assay kit (BioVision Incorporated, Milpitas, CA, USA) according to manufacturer instructions; absorbance was read at 532 nm. Adipogenesis was measured by quantifying triglyceride accumulation in muscle tissue. The sub-epidermal adipose tissue amounts were analyzed using an Adipogenesis Assay kit (Abnova, Taipei City, Taiwan); absorbance was read at 570 nm.

### Statistical analysis

All data were analyzed by one-way analysis of variance (ANOVA) using the SAS/STAT software (SAS, 1988). If significant differences were detected (p < 0.05), means of dependent variables were compared using Tukey’s honestly significant difference (HSD) test. Our data were represented as mean ± SEM.

## Results

### Growth performance, nutrient utilization and proximate composition of shrimp carcass

Growth performance parameters were not significantly different (p > 0.05) among the treatments, although FBW was numerically higher in shrimps fed with the diets 4, 6, 7, and 8 compared to the other diets (Table 3). Similarly, the feed efficiencies, including FCR, PER, PPV and LPV were also not significantly different among treatments. Differences in survival rate among treatments were not significant (Table 3).

**Table 3:** Growth performance and feed efficiencies of shrimp*.

	Experimental diets							
	1	2	3	4	5	6	7	8
FBW (g)	20.74 ± 0.47^cd^	21.13 ± 0.66^bcd^	20.65 ± 0.29^cd^	23.10 ± 0.52^a^	19.90 ± 0.28^d^	21.84 ± 0.65^abc^	22.49 ± 0.47^ab^	22.89 ± 0.47^a^
BMG (%)	562.24 ± 27.28	578.05 ± 20.49	589.15 ± 96.44	612.76 ± 50.94	531.83 ± 90.16	626.37 ± 25.15	627.35 ± 59.07	636.91 ± 21.20
FCR	2.16 ± 0.09	2.04 ± 0.12	2.18 ± 0.13	2.38 ± 0.29	2.35 ± 0.33	2.28 ± 0.30	2.36 ± 0.18	2.07 ± 0.07
PER	0.91 ± 0.05	1.02 ± 0.05	0.96 ± 0.05	0.91 ± 0.13	0.91 ± 0.12	0.92 ± 0.11	0.87 ± 0.07	0.97 ± 0.03
PPV (%)	24.4 ± 2.21	27.3 ± 2.85	25.5 ± 1.75	25.1 ± 3.92	25.9 ± 1.85	23.7 ± 2.58	23.5 ± 1.22	26.1 ± 1.15
LPV (%)	20.40 ± 1.65	22.33 ± 3.28	23.04 ± 1.28	22.32 ± 2.43	19.96 ± 1.78	21.43 ± 2.09	19.28 ± 1.21	22.60 ± 1.46
Survival (%)	90.67 ± 3.53	85.33 ± 3.53	82.67 ± 5.81	73.33 ± 3.53	90.67 ± 1.33	82.67 ± 2.67	80.00 ± 4.00	86.67 ± 5.81

*Values are mean ± SE of three replicates per group. Means in the same row with different superscripts are significantly (p < 0.05) different.

The moisture and ash contents were significantly higher (p < 0.05) for shrimp fed the control diet (diet 1) compared to all other treatments (Table 4). Crude protein content was significantly higher (p < 0.05) in those shrimps fed with the diets 4 and 5 than all others except for shrimp fed diet 7. Meanwhile, crude lipid was not significantly different (p > 0.05) among the treatments but was slightly lower for shrimp fed the control diet.

**Table 4:** Proximate composition (g/kg) of the whole carcass of shrimp*.

	Experimental diets							
	1	2	3	4	5	6	7	8
Moisture	782.3 ± 3.2^a^	757.7 ± 3.0^b^	757.3 ± 2.7^b^	755.0 ± 2.7^b^	753.7 ± 4.7^b^	759.3 ± 1.2^b^	755.0 ± 2.5^b^	754.0 ± 0.3^b^
Crude protein	213.0 ± 3.2^b^	213.0 ± 2.5^b^	211.0 ± 8.8^b^	222.7 ± 8.7^a^	230.7 ± 4.6^a^	210.0 ± 5.4^b^	216.3 ± 1.8^ab^	212.7 ± 6.8^b^
Crude lipid	17.8 ± 2.4	16.8 ± 4.6	20.0 ± 2.5	16.3 ± 5.6	15.2 ± 2.0	13.4 ± 3.5	14.3 ± 3.4	19.2 ± 2.5
Crude ash	16.1 ± 0.9^a^	12.6 ± 2.2^b^	14.1 ± 0.5^b^	14.5 ± 0.5^b^	14.2 ± 1.5^b^	15.3 ± 0.9^b^	15.1 ± 1.7^b^	14.0 ± 0.8^b^

^*^Values are mean ± SE of three replicates per group. Means in the same row with different superscripts are significantly (p < 0.05) different.

### Tail muscle fatty acids composition

**Figure 1:**
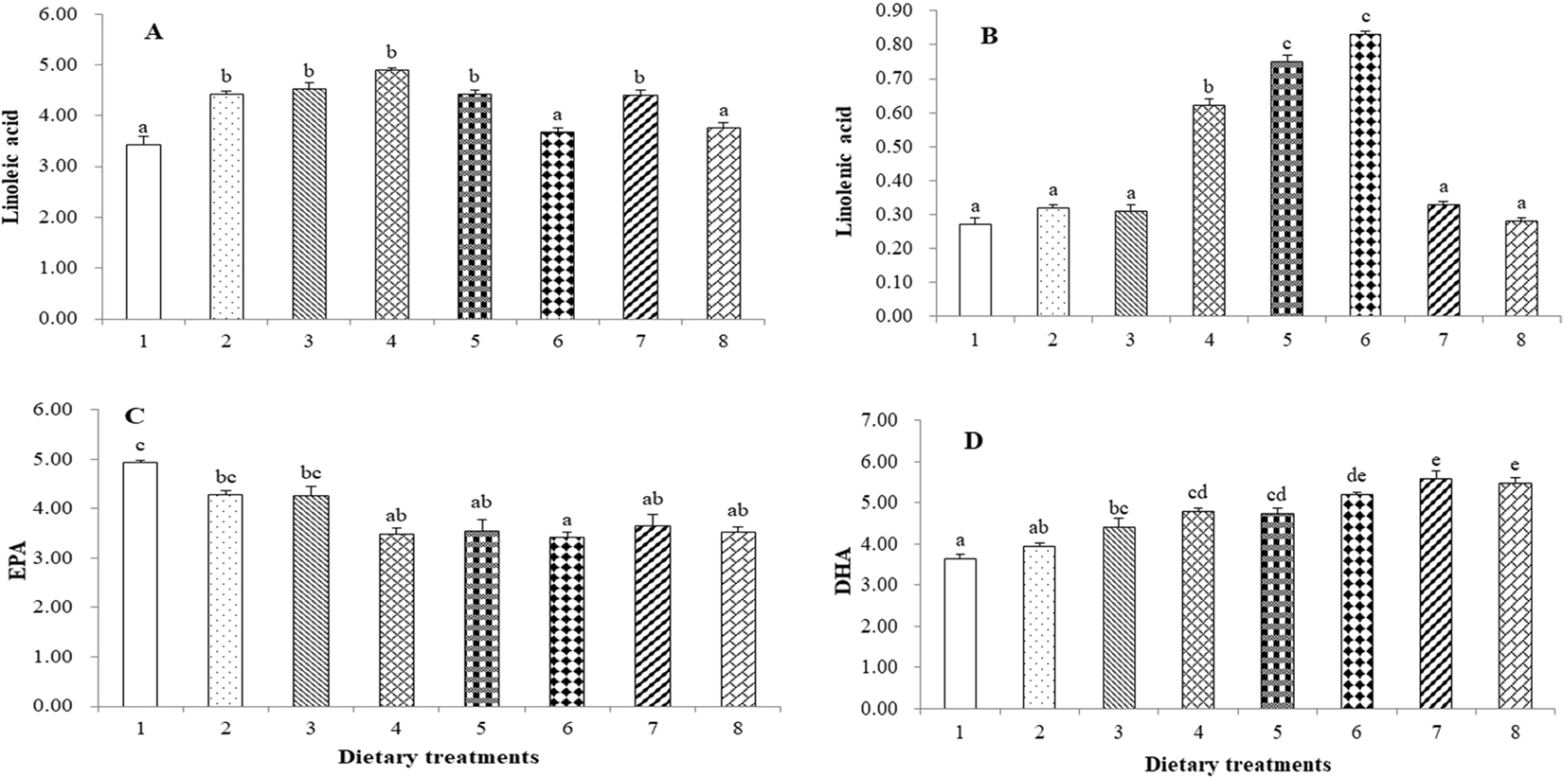
Linoleic acid (**A**), Linolenic acid (**B**), EPA (**C**) and DHA (**D**) compositions (% of total lipids) in the tail muscle of shrimp. Values are mean ± SE of three replicates per group. Different letters above each bar indicate significant differences (p < 0.05).

The tail muscle fatty acids composition is shown in Table 5. Among the saturated fatty acids (SFA), palmitic acid (C16) was significantly higher in shrimp those fed with the diets 6–8 than those fed diets 1–4, while shrimp fed that diets 5–8 had significantly lower lignoceric acid (C24) levels than those fed diets 1–3. There was no significant difference for the total SFA level among the treatments. Among the monounsaturated fatty acids (MUFA), C16:1n-7 was lower (p < 0.05) in shrimp fed diet 4 than those fed diets 1, 6, 7, or 8, while oleic acid (C18:1n-9) in the muscle was higher (p < 0.05) in shrimp fed diet 1 than all others except in shrimp fed with diet 2. Meanwhile, shrimp fed diet 1 had significantly higher (p < 0.05) gondoic acid (C20:1n-9) than those fed all other diets; the lowest amount was in shrimp fed diets 5, 6, 7, and 8. Shrimp fed with diets 1, 6, or 8 had significantly lower (p < 0.05) LA than all others (Fig. 1A), while αALA was higher (p < 0.05) in shrimp fed diets 5 and 6 than all other treatments (Fig. 1B). There was significant influence of dietary treatments on the total MUFA level, and the highest level was observed in the group fed diet 1 trailed by diets 2 and 3, respectively. Treatments 2–5 displayed higher total n-6 PUFA level compared to the control (fish oil) and groups 6–8. The total n-3 PUFA level was unaffected by the experimental diets (Table 5). Among the LC-PUFAs, EPA decreased with increasing amounts of microalgae meal in the diets (Fig. 1C), while DHA significantly increased with higher dietary inclusions of algae meal (Fig. 1D). The total LC-PUFA level was unaffected. Shrimp fed diets 6, 8, and 1 displayed the highest n-3 and n-6 ratio compared to those fed with other diets, respectively (Table 5).

**Table 5:** Fatty acids (% of total lipid) from the tail muscle of shrimp*.

Fatty acids	Experimental diets							
	1	2	3	4	5	6	7	8
C6	0.46 ± 0.03	0.42 ± 0.01	0.51 ± 0.08	0.41 ± 0.00	0.42 ± 0.00	0.47 ± 0.04	0.42 ± 0.00	0.47 ± 0.04
C8	0.53 ± 0.01	0.67 ± 0.11	0.53 ± 0.00	0.49 ± 0.02	0.54 ± 0.00	0.51 ± 0.02	0.54 ± 0.00	0.51 ± 0.02
C10	0.37 ± 0.01	0.42 ± 0.03	0.39 ± 0.02	0.38 ± 0.02	0.38 ± 0.02	0.37 ± 0.01	0.37 ± 0.01	0.39 ± 0.02
C11	0.47 ± 0.01	0.41 ± 0.04	0.44 ± 0.04	0.40 ± 0.03	0.45 ± 0.04	0.43 ± 0.03	0.41 ± 0.04	0.48 ± 0.01
C13	0.49 ± 0.00	0.49 ± 0.00	0.49 ± 0.00	0.49 ± 0.00	0.49 ± 0.00	0.49 ± 0.00	0.49 ± 0.00	0.49 ± 0.00
C14	0.19 ± 0.03	0.15 ± 0.02	0.14 ± 0.01	0.14 ± 0.01	0.14 ± 0.00	0.16 ± 0.00	0.16 ± 0.01	0.16 ± 0.00
C15	0.11 ± 0.01	0.11 ± 0.00	0.10 ± 0.00	0.11 ± 0.01	0.11 ± 0.00	0.12 ± 0.00	0.12 ± 0.01	0.12 ± 0.00
C16	5.94 ± 0.07^b^	6.04 ± 0.13^b^	6.38 ± 0.39^b^	6.48 ± 0.13^b^	6.70 ± 0.22^ab^	7.08 ± 0.08^a^	7.21 ± 0.21^a^	7.07 ± 0.05^a^
C17	0.29 ± 0.01	0.31 ± 0.00	0.30 ± 0.01	0.31 ± 0.02	0.31 ± 0.02	0.32 ± 0.01	0.33 ± 0.01	0.31 ± 0.00
C18	3.09 ± 0.02	3.50 ± 0.03	3.39 ± 0.16	3.43 ± 0.13	3.35 ± 0.18	3.39 ± 0.15	3.53 ± 0.16	3.36 ± 0.11
C23	24.86 ± 0.06	24.74 ± 0.03	24.71 ± 0.08	24.60 ± 0.07	24.66 ± 0.04	24.59 ± 0.03	24.81 ± 0.09	24.80 ± 0.12
C24	0.26 ± 0.02^a^	0.21 ± 0.00^b^	0.18 ± 0.01^bc^	0.16 ± 0.01^cd^	0.13 ± 0.00^d^	0.13 ± 0.00^d^	0.14 ± 0.01^d^	0.14 ± 0.01^d^
C16:1n-7	0.59 ± 0.07^a^	0.41 ± 0.02^bc^	0.38 ± 0.02^bc^	0.34 ± 0.01^c^	0.37 ± 0.04^bc^	0.49 ± 0.01^ab^	0.46 ± 0.02^abc^	0.57 ± 0.04^a^
C18:1n-9	4.70 ± 0.22^a^	4.17 ± 0.11^ab^	3.84 ± 0.13^bc^	3.33 ± 0.08^cd^	2.98 ± 0.09^d^	3.05 ± 0.08^d^	3.32 ± 0.19 ^cd^	2.95 ± 0.15^d^
C18:2n-6	3.42 ± 0.18^b^	4.42 ± 0.07^a^	4.52 ± 0.13^a^	4.89 ± 0.05^a^	4.41 ± 0.10^a^	3.68 ± 0.08^b^	4.40 ± 0.10^a^	3.75 ± 0.12^b^
C18:3n-3	0.27 ± 0.02^c^	0.32 ± 0.01^c^	0.31 ± 0.02^c^	0.62 ± 0.02^b^	0.75 ± 0.02^a^	0.83 ± 0.01^a^	0.33 ± 0.01^c^	0.28 ± 0.01^c^
C20:1n-9	0.83 ± 0.03^a^	0.49 ± 0.02^b^	0.37 ± 0.02^c^	0.16 ± 0.01^d^	0.14 ± 0.01^d^	0.14 ± 0.01^d^	0.16 ± 0.01^d^	0.15 ± 0.01^d^
C20:2n-6	0.38 ± 0.03^bc^	0.50 ± 0.02^ab^	0.51 ± 0.02^a^	0.48 ± 0.03^ab^	0.37 ± 0.04^bc^	0.33 ± 0.02^c^	0.41 ± 0.03^abc^	0.33 ± 0.02^c^
C20:3n-3	0.90 ± 0.03^d^	1.06 ± 0.02^cd^	1.20 ± 0.05^bc^	1.25 ± 0.02^bc^	1.33 ± 0.02^ab^	1.50 ± 0.07^a^	1.47 ± 0.07^a^	1.51 ± 0.02^a^
C20:5n-3	4.94 ± 0.04^a^	4.27 ± 0.10^ab^	4.26 ± 0.18^ab^	3.49 ± 0.12^bc^	3.54 ± 0.24^bc^	3.42 ± 0.11^c^	3.64 ± 0.25^bc^	3.53 ± 0.09^bc^
C22:6n-3	3.64 ± 0.12^e^	3.94 ± 0.08^de^	4.41 ± 0.21^cd^	4.78 ± 0.08^bc^	4.72 ± 0.16^bc^	5.21 ± 0.05^ab^	5.58 ± 0.19^a^	5.46 ± 0.15^a^
*Major groups*								
Σ SFA	37.07 ± 0.33	37.46 ± 0.26	37.55 ± 0.67	37.38 ± 0.17	37.66 ± 0.39	38.06 ± 0.33	38.53 ± 0.32	38.31 ± 0.24
Σ MUFA	6.13 ± 0.32^d^	5.07 ± 0.14^c^	4.59 ± 0.15^bc^	3.83 ± 0.08^ab^	3.49 ± 0.10^a^	3.68 ± 0.09^a^	3.93 ± 0.21^ab^	3.67 ± 0.18^a^
Σ n-6 PUFA	3.79 ± 0.20^a^	4.92 ± 0.05^b^	5.03 ± 0.15^b^	5.37 ± 0.06^b^	4.78 ± 0.13^b^	4.01 ± 0.10^a^	4.81 ± 0.13^a^	4.08 ± 0.10^a^
Σ n-3 PUFA	9.76 ± 0.10	9.59 ± 0.04	10.18 ± 0.43	10.14 ± 0.20	10.33 ± 0.44	10.97 ± 0.20	11.02 ± 0.49	10.78 ± 0.13
Σ LC-PUFA	9.86 ± 0.10	9.76 ± 0.05	10.39 ± 0.45	10.00 ± 0.23	9.95 ± 0.43	10.47 ± 0.22	11.10 ± 0.52	10.83 ± 0.15
n-3/n-6	2.58 ± 0.11^cd^	1.95 ± 0.01^a^	2.02 ± 0.03^ab^	1.89 ± 0.05^a^	2.16 ± 0.08^ab^	2.73 ± 0.02^d^	2.29 ± 0.04^bc^	2.64 ± 0.08^d^

^*^Values are mean ± SE of three replicates per group. Means in the same row with different superscripts are significantly (p < 0.05) different.

### Tail muscle sub-epidermal adipose tissue, and total cholesterol contents, intestinal lipase activity, lipid peroxidation and antioxidant enzymes activities

**Figure 2:**
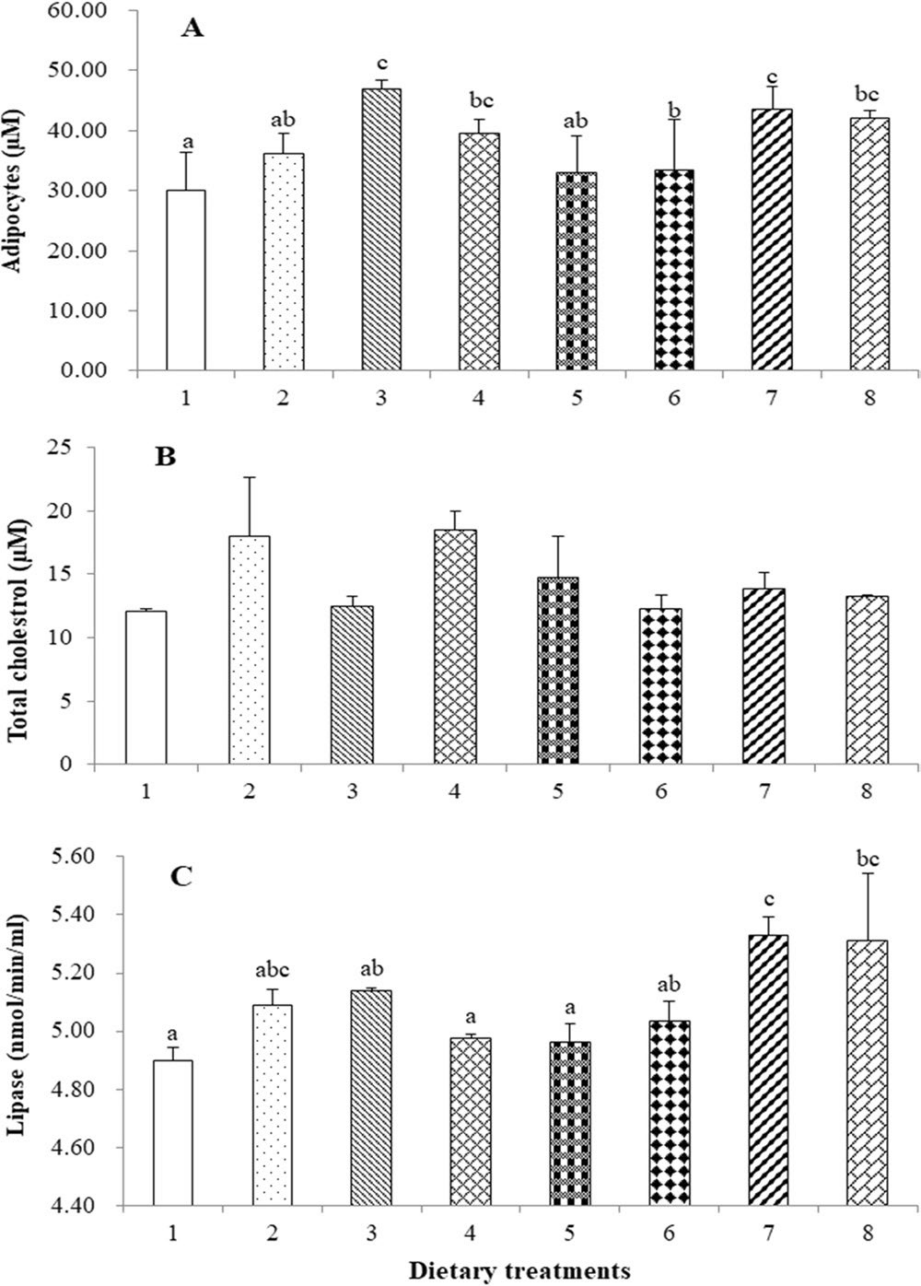
Total adipocyte counts (**A**), and total cholesterol content in the tail muscle (**B**) and intestinal lipase activity (**C**) of shrimp. Values are mean ± SE of three replicates per group. Different letters above each bar indicate significant differences (p < 0.05).

**Figure 3:**
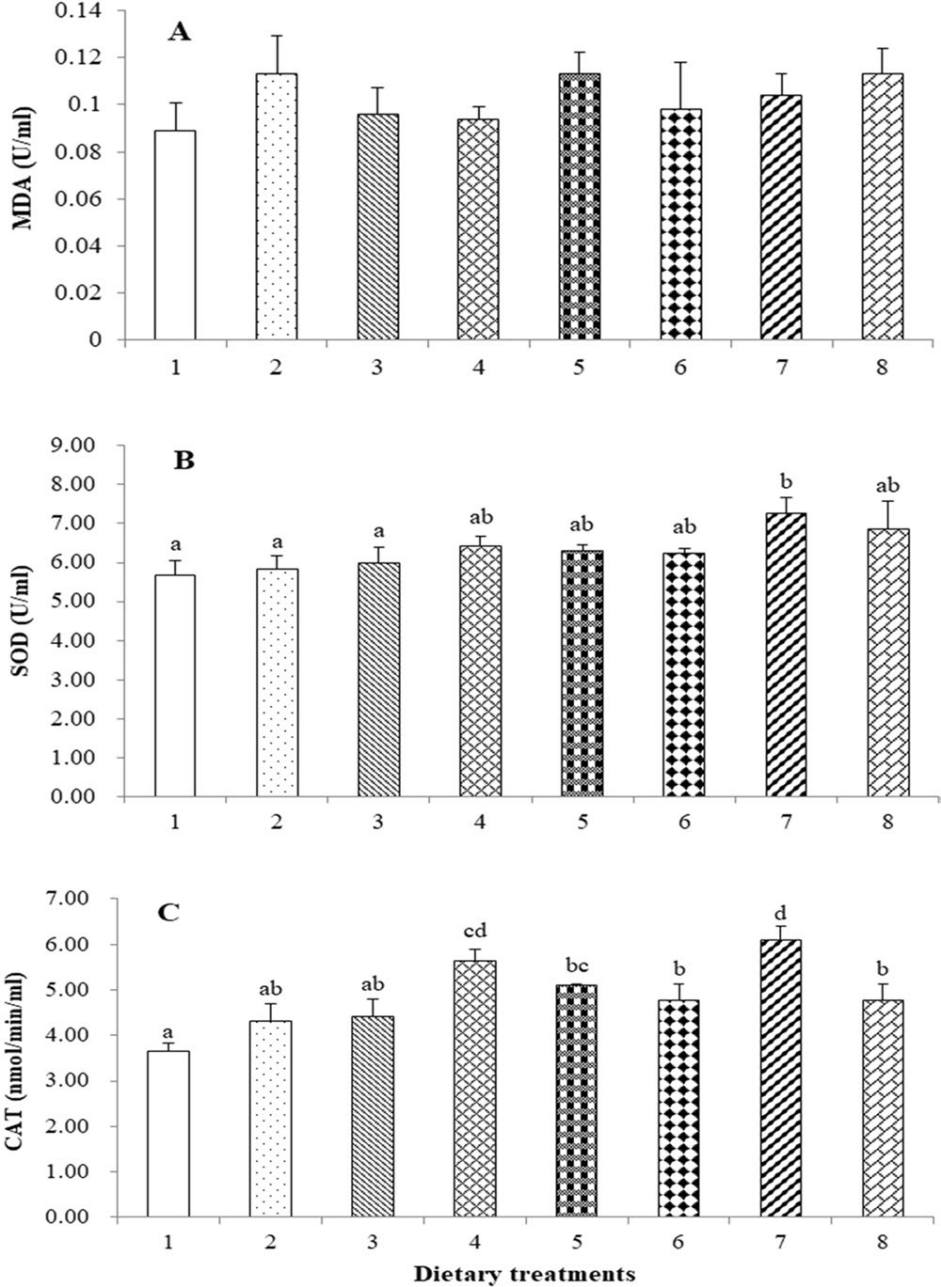
Malondialdehyde (MDA) content (**A**) and activities of superoxide dismutase (SOD) (**B**) and catalase (CAT) (**C**) in the tail muscle of shrimp. Values are mean ± SE of three replicates per group. Different letters above each bar indicate significant differences (p < 0.05).

The total adipocyte count in the tail muscle was significantly higher in shrimp those fed with diets 3 and 7 compared to those fed diets 1, 2, 5 and 6; shrimp fed diet 1 had the lowest adipocyte count (Fig. 2A). The total cholesterol levels of the shrimp were unaffected by the dietary treatment (Fig. 2B). Intestinal lipase activity was lowest for shrimp fed diets 1, 4 and 5 and significantly lower than shrimp fed diets 7 or 8 (Fig. 2C). The amount of MDA was unaffected among the different treatments (Fig. 3A). SOD activity in shrimp fed diet 7 was significantly higher than those fed with diets 1, 2, or 3 (Fig. 3B). Similarly, CAT activity was significantly higher in shrimp those fed diet 7 than all other diets, except diet 4 (Fig. 3C).

## Discussion

The results of the present study indicated that *Schizochytrium* meal fed at different levels or in combinations with SBO and/or LSO as a replacer to FO did not cause a significant difference in the growth or feed efficiency of shrimp. Moreover, there were no indications of feed rejection when shrimp were fed diets without FO and shrimp appeared healthy throughout the feeding trial. In fact, shrimp fed diets with the highest amounts of the microalgae meal tended to have slightly higher growth than those fed the control diet with only FO as the added lipid source although differences were not statistically significant. In addition, the tail muscle fatty acids composition had significantly higher amounts of LC-PUFA compared to shrimp fed the control diet. FO can be completely replaced with SBO and LSO in the diet of *L. vannamei* by balancing the omega-3/omega-6 ratios^18^. Recently, Kumar *et al*.^19^ have used the algal (*Schizochytrium* sp.) meal as a fish oil replacer in fish meal free diets for shrimp, however the in the current study we have used the algae meal as a substitute to fish oil, but our experimental diets were fish meal-based diets (25% fish meal). Kumar *et al*.^19^ observed that replacement of fish oil with algal meal and vegetable oil increased the weight of shrimp and nutritional value (n-3 PUFA and LC-PUFA significantly increased) of shrimp muscle. Algal meal combined with vegetable oil could replace 75% fish oil in shrimp diets. Recently, a combination of SBO, LSO, and beef tallow led to significantly better growth in *L. vannamei* compared to FO only^20^. Nevertheless, the use of terrestrially-based oils substantially reduces the LC-PUFA content^20,21^, which is less healthy for the human consumer. The use of algae meal, including *Schizochytrium* sp. and *Mortierella* sp., are a viable replacement for FO in the diets of *L. vannamei*, that maintain or even enhance the LC-PUFA content^6,10–13^. This is attributed to the high LC-PUFA content of the microalgae meals used, but the oil from *Schizochytrium* sp. had relatively high and low levels of DHA and EPA, respectively. An imbalance in these fatty acids in other microalgae meals was implicated as a potential reason for lower growth in the shrimp, *Litopenaeus schmitti*^22^. This is consistent with the concept that a monoculture of microalgae is inferior compared to the use of microalgae mixtures when compensating for any potential nutritional deficiencies to various aquatic animals^23,24^. In this study, *L. vannamei* appeared to utilize *Schizochytrium* sp. well despite the much higher DHA levels relative to EPA.

Due to lower EPA levels in *Schizochytrium* sp., diets 4–6 were formulated with LSO, which contains relatively high amounts of ALA, which is a precursor to EPA synthesis. This was done since *L. vannamei* has shown some ability for elongation and desaturation, although this ability is depressed when shrimp are fed with high levels of either ALA or LA^20^. This is believed to be due to a feed-back mechanism^20^. Indeed, in this study, dietary LSO had no effect on muscle EPA content of the shrimp. This finding is in general agreement with other studies showing the fatty acid composition of *L. vannamei* reflects that of the diet^18,21,25,26^. In addition, dietary treatments significantly influenced the major fatty acids of the tail muscle (total MUFA, n-6 PUFA and ratio of n-3/n-6) of Pacific white shrimp in this study. Previous study suggested that for Pacific white shrimp production a range of lipid sources can be used for growth but a lipid source containing high levels of n-3 HUFA and high n-3/n-6 ratios are preferred for the shrimp being used for human consumption^18^. In this study, shrimp fed with diets 6 and 8 followed by diet 1 displayed the highest n-3/n-6 ratios in the tail muscle compared to those fed with other diets. Therefore, our findings indicate that *Schizochytrium* sp. meal could be used as better source than fish oil for shrimp feed because it provides better and healthier shrimp product for human food.

Fatty acids with a high degree of saturation are more prone to lipid peroxidation. Despite the differences in the fatty acid composition among the shrimp, with the lowest and highest content of LC-PUFA in shrimp fed with diets 2 and 8, respectively, the amount of MDA in the tail muscle was unaffected by the diets. This may be due to fact that SOD and CAT activities were not disrupted, since these enzymes are primarily responsible for protecting animals from oxidative stress^27^. In fact, SOD was significantly higher only for shrimp fed with diet 7, which corresponded to the highest CAT activity. A similar finding of enhanced antioxidant enzyme activity was observed in *L. vannamei* those fed *Schizochytrium* sp., along with improved innate immunity and resistance to *V. harveyi*^13^. Therefore, the higher muscle SOD and CAT activities in the current study may also indicate an improvement in the anti-oxidative status of the shrimp rather than a stress response. This also appears to be supported based on the fact that hemolymph parameters of the shrimp (including albumin, glucose, total protein, and globulin), which can indicate stress^28^, were unaffected in this study (data not reported). When microalgae meal was included at the highest level (we tested) and was the only oil source, no improvement to SOD and CAT was found. This may indicate that the levels used were excessive, but it requires further investigation.

One of the potential ways to determine the nutritional status of animals is the proximate composition of the animal including the adipocyte counts, which is a measurement of the fat content. Limited information is available on measuring adipocytes in aquatic animals, but adipocyte counts showed a similar pattern to the crude lipid content of the shrimp carcass. However, only adipocyte counts were affected by diet (with significantly higher counts in shrimp fed with diets 3 and 7). An increase in adipocyte counts could potentially be due to improved energy reserves or decreased lipid digestion. In terms of the latter, fatty acid digestion is greatly influenced by the carbon length and degree of saturation in the black tiger shrimp, *Penaeus monodon*^29^. LC-PUFA generally tends to have a lower digestibility than SFA and MUFA, which further decreases with increasing carbon length^29^. However, the high adipocyte counts found in shrimp fed diet 3 did not show any pattern related to carbon length, saturation, or any unique fatty acid type, group or ratio. In addition, for shrimp those fed with diet 3, the higher number of sub-epidermal adipose tissue was not linked to growth. Additional investigations are required to better elucidate the cause for higher adipocyte counts in diet 3.

Alternatively, the high DHA content in shrimp those fed with diets 7 and 8 may have led to a lower digestibility and, thus, an accumulation of lipids. A decrease in digestibility is consistent with the high intestinal lipase activity found in shrimp fed both of these diets, but particularly in shrimp fed diet 8, which contained the highest amount of DHA. Crustaceans have the ability to modify their digestive enzymes as demonstrated in redclaw crayfish, *Cherax quadricarinatus*^30^, and *L. vannamei*^31^. For example, the inclusion of dietary plant-based ingredients, such as sorghum, canola meal, brewer’s yeast, soybean meal, or lupin meal have shown increased lipase activity in the hepatopancreas and midgut of *C. quadricarinatus*^30^, whereas lipase activity was decreased in *L. vannamei* those fed with increasing levels of yeast extract^31^. However, increasing dietary *Schizochytrium* meal at the expense of FO had no effect on intestinal/hepatopancreatic leucine aminopeptidase or alkaline phosphatase activity or on hepatopancreatic trypsin or amylase activity in *L. vannamei* larvae, which can be interpreted as the microalgae having no adverse effect on their nutritional status^10^.

In addition to shrimp tending to have higher lipid content in the diets with the highest levels of *Schizochytrium* meal, all shrimp fed a diet containing some microalgae meal had significantly lower moisture and ash content than those fed the control diet. This finding might indicate an improvement in osmoregulation; since at elevated salinities, shrimp need to remove excess minerals. Indeed, the use of high levels of dietary LC-PUFA improves osmoregulation at both low and high salinities in various crustaceans^32,33^, including *L. vannamei*^34^. Some of the potential mechanisms by which this may occur include a more fluid cell membrane that would improve enzymatic activities, greater gill surface area, and possibly better overall nutritional status^34^. This deserves further research since *L. vannamei* can be farmed in various salinity conditions, although a particular focus on low salinity waters would be beneficial since the production of shrimp in inland (low salinity waters) has become an emerging industry around the world^20^. It is also important to note that microalgae meal contains not only oil, but also other bioactive compounds, including essential amino acids, vitamins, pigments, phenolics, polyamines, etc.^35^. Therefore, it is possible that the physiologic responses of shrimp in this study, including growth, enzyme activity, lipid peroxidation, and proximate composition might also be influenced by any or all of these factors and requires further specific molecular study.

## Conclusion

In conclusion, *Schizochytrium* sp. meal can be used to replace completely FO without compromising growth. Among the diets tested in this study, the diet containing second highest level microalgae meal combined with SBO provided the best benefits in terms of slightly improved growth, and significantly higher antioxidant enzyme activity, lipase activity, and LC-PUFA content as well as indications of more available energy. Moreover, Pacific white shrimp displayed higher proportions of n-3/n-6 in treatment 6 (algae meal and LSO based diet) and 8 (only algae meal based diet as lipid source) in the tail muscle, which is healthier sea food preferred for human consumption. This study, therefore, demonstrates that *Schizochytrium* sp. meal can be superior to the traditional ingredient, FO, in the diets of *L. vannamei* which provided improved shrimp quality as a healthier food. Considering the relatively high cost of *Schizochytrium* sp. meal, further research into optimal ratios with SBO could be worthwhile to improve feed cost-effectiveness.
